# Human skin equivalents cultured under hypoxia display enhanced epidermal morphogenesis and lipid barrier formation

**DOI:** 10.1038/s41598-019-44204-4

**Published:** 2019-05-24

**Authors:** Arnout Mieremet, Adela Vázquez García, Walter Boiten, Rianne van Dijk, Gert Gooris, Joke A. Bouwstra, Abdoelwaheb El Ghalbzouri

**Affiliations:** 1 Department of Dermatology, Leiden University Medical Center, Leiden, The Netherlands; 2 Division of BioTherapeutics, Leiden Academic Centre for Drug Research, Leiden University, Leiden, The Netherlands

**Keywords:** Skin models, Tissue engineering

## Abstract

Human skin equivalents (HSEs) are three-dimensional cell models mimicking characteristics of native human skin (NHS) in many aspects. However, a limitation of HSEs is the altered *in vitro* morphogenesis and barrier formation. Differences between *in vitro* and *in vivo* skin could have been induced by suboptimal cell culture conditions, of which the level of oxygen *in vitro* (20%) is much higher than *in vivo* (0.5–8%). Our aim is to study how external oxygen levels affect epidermal morphogenesis and barrier formation in HSEs. In the present study, fibroblast and keratinocyte monocultures, and HSEs were generated under 20% (normoxia) and 3% (hypoxia) oxygen level. In all cultures under hypoxia, expression of hypoxia-inducible factor target genes was increased. Characterization of HSEs generated under hypoxia using immunohistochemical analyses of morphogenesis biomarkers revealed a reduction in epidermal thickness, reduced proliferation, similar early differentiation, and an attenuated terminal differentiation program compared to normoxia, better mimicking NHS. The stratum corneum ceramide composition was studied with liquid chromatography coupled to mass spectrometry. Under hypoxia, HSEs exhibited a ceramide composition that more closely resembles that of NHS. Consequently, the lipid organization was improved. In conclusion, epidermal morphogenesis and barrier formation in HSEs reconstructed under hypoxia better mimics that of NHS.

## Introduction

Three-dimensional (3D) human skin equivalents (HSEs) are mainly used for toxicology screenings and for research purposes to increase the knowledge on skin biology, or particularly the skin barrier formation. Various types of 3D skin models are currently available, including epidermal skin models, full thickness skin models (FTMs) **(**Supplementary Fig. 1a,b), explant models, and skin-on-a-chip models, listed by increased complexity and physiological relevance^1–7^. As reviewed recently by Niehues *et al*.^8^, while all of these models have advantages and drawbacks, a known limitation of the *in vitro* developed skin models is the altered barrier formation and resulting reduced functionality when compared to native human skin (NHS)^9–11^. This could lead to inadequate *in vitro – in vivo* correlations regarding pharmacokinetics and permeability testing of compounds or misinterpretation of adverse outcome pathways^12^.

The permeability barrier of the epidermis resides mainly in the uppermost layer, the stratum corneum (SC). In this layer of dead cells, corneocytes are keratinized and cross-linked to form impermeable structures^13,14^. In between the corneocytes, the lipids form the only continuous pathway through the SC (Supplementary Fig. 1c,d). The lipids form a highly structured matrix organized in densely packed crystalline lamellar phases^15^ (Supplementary Fig. 1e,f). This lipid matrix contributes substantially to the barrier functions of the skin, which are protecting from external substance or allergen entry and restricting water loss^15,16^. The most abundant lipid classes within the SC are cholesterol, free fatty acids (FFAs) and ceramides^17^. Several ceramide subclasses exist, categorized based on their head group architecture^18–21^. When considering the total ceramides (CERs_total_) subclasses analyzed in this study, these consist of ceramides (CERs) and acylceramides (CERs EO) (Supplementary Fig. 2). Alterations in lipid composition most probably lead to an altered organization and can thereby reduce the permeability barrier functionality. This is encountered in HSEs and diseased skin conditions^22,23^. In detail, the alterations in the lipid composition of HSEs as compared to NHS include an altered CERs_total_ subclass profile, reduction of CER carbon chain length, and a higher level in unsaturation. These alterations directly affect the lipid organization, including the reduction in lamellar phase repeat distance and the conversion in lateral packing from a predominant orthorhombic to a hexagonal packing^22,24^.

Normalization of the barrier formation of HSEs is a complex procedure, as many external factors differ between *in vivo* and *in vitro* culture conditions. Besides a nutrient imbalance, several external factor differences are encountered, such as the constant high temperature (37 °C) *in vitro*, the lack of sunlight, a high environmental humidity, and a high oxygen level. Since the epidermis is not directly supplied with blood, the oxygen levels in the epidermis are regarded as low, ranging from 0.5% to 8% *in vivo*^25,26^. In contrast, during culturing of HSEs the oxygen saturation is corresponding to the atmospheric oxygen pressure of the incubator (±20%)^27^.

Oxygen levels affect cellular metabolism, as these are sensed continuously and can activate a signaling cascade mediated by Hypoxia-Inducible Factors (HIFs)^28^. HIFs are highly conserved transcriptional complexes comprising an oxygen labile subunit (HIF-1α, HIF-2α or HIF-3α) and a constitutively nuclear expressed HIF-1β subunit which can bind after heterodimerization to hypoxia response elements (HREs)^29^. As reported by Seleit *et al*.^30^, in normal conditions HIF-1α is expressed in the basal and suprabasal layers in 90% of normal skin biopsies. During low or mild oxygen, nuclear translocation of HIF-α occurs, leading to transcriptional activation of HIF target genes^31^. More than 200 genes are involved in this pathway, of which many are involved in processes such as glycolysis, angiogenesis, apoptosis, adhesion, migration, and metastasis^26,28^. Previous *in vivo* murine studies have already revealed that the HIF pathway is a crucial determinant of skin homeostasis^32,33^.

Because of the broad repercussion of HIFs in cellular gene expression, also of those involved in terminal differentiation and barrier properties of keratinocytes, it is of importance to obtain detailed insights on the effect of oxygen levels during the development of HSEs. Therefore, we aim to study whether a reduction from a normoxic (20%) to a hypoxic (3%) oxygen level leads to higher resemblance to native epidermal morphogenesis and barrier formation in HSEs. Herein, we show that reduction from normoxia to hypoxia improved epidermal morphogenesis and SC barrier lipid composition mediated by activated HIF signaling pathway in HSEs.

## Results

### Fibroblast and keratinocyte monocultures under hypoxia

**Figure 1:**
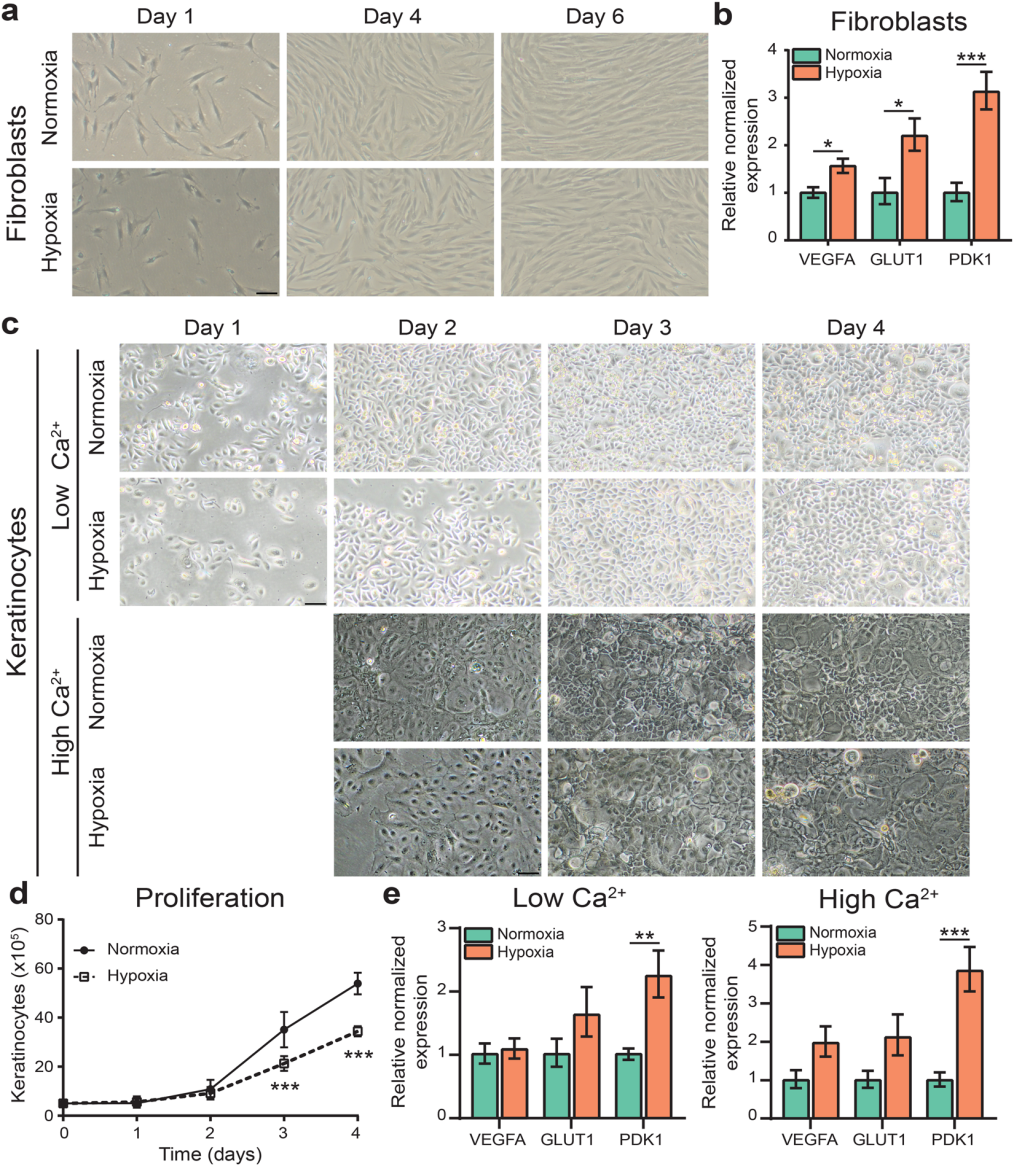
Fibroblast and keratinocyte monocultures under hypoxia. (**a**) Phase contrast images of fibroblasts under normoxia (20%) and hypoxia (3%) at indicated period. (**b**) Gene expression of HIF target genes in fibroblasts cultured under hypoxia and normoxia, indicated by mean ± s.e.m., n = 4. (**c**) Phase contrast images of keratinocytes cultured for 4 days in low (proliferating) calcium medium and of keratinocytes cultured for 1 day in low calcium medium and 3 days in high (differentiating) calcium medium under normoxia or hypoxia. (**d**) Proliferation of keratinocytes in low calcium culture medium under hypoxia, indicated by mean ± 95% confidence interval. (**e**) Expression of HIF target genes in keratinocytes cultured in low or high calcium medium in hypoxia as compared to normoxia, indicated by mean ± s.e.m., n = 4. Statistical differences are noted as *, ** or ***, corresponding to P < 0.05, <0.01, <0.001. Scale bar indicates 50 µm.

To fully understand the effects of oxygen levels on skin cell cultures, we initially characterized monocultures of fibroblasts or keratinocytes developed at 20% O_2_ for normoxia or 3% O_2_ for hypoxia. The spindle shaped morphology of fibroblast monocultures was unaffected after 6 days in culture under hypoxia when compared to normoxia (Fig. 1a). Next, we performed gene expression analysis on pre-selected HIF target genes involved in metabolic reprogramming (glucose transporter 1 (GLUT1) and pyruvate dehydrogenase lipoamide kinase isozyme 1 (PDK1)) and the angiogenic molecule vascular endothelial growth factor A (VEGFA). Significant upregulation of these HIF target genes was detected in fibroblasts when cultured under hypoxia (Fig. 1b). To investigate the effect of oxygen levels on keratinocyte growth and differentiation, these were cultured in low (proliferating) and high (differentiating) calcium (Ca^2+^) medium for several days in normoxia and hypoxia **(**Fig. 1c**)**. Similar to fibroblasts, keratinocyte morphology was not altered when cultured under hypoxia. However, keratinocytes showed a significant decrease in cell proliferation rate under hypoxia from 3 days in monoculture (Fig. 1d). Furthermore, gene expression analysis of subconfluent proliferating and confluent differentiating keratinocytes also showed upregulation of the pre-selected HIF target genes under hypoxia (Fig. 1e). This indicates that reduction from 20% to 3% oxygen level is sufficient to induce adaptation and metabolic reprogramming of fibroblasts and keratinocytes to hypoxic conditions.

### Human skin equivalents generated under hypoxia

**Figure 2:**
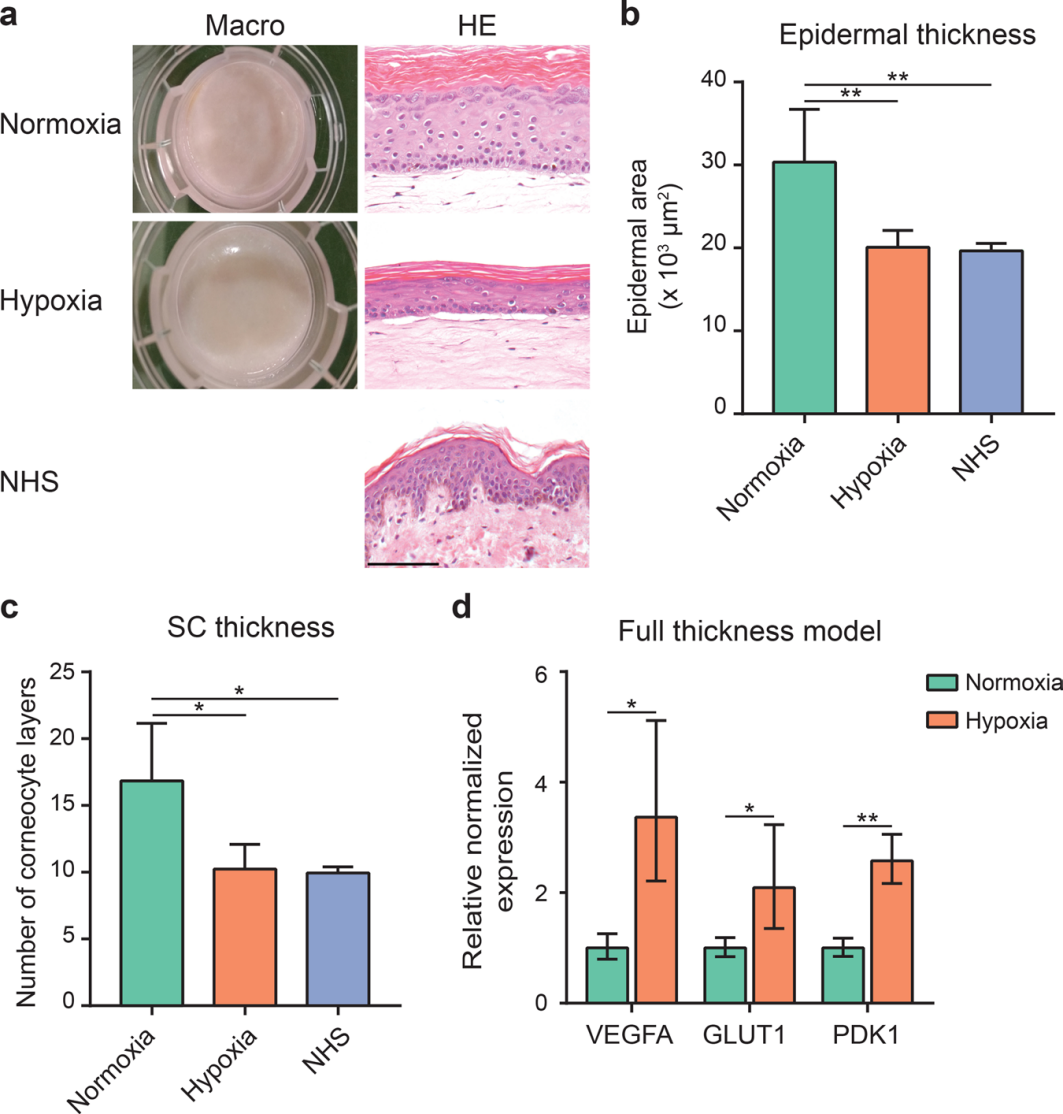
Full thickness models generated under hypoxia. (**a**) Macroscopic images of FTMs generated under normoxia or hypoxia. General morphology assessed through hematoxylin and eosin (HE) stained cross sections of FTMs and NHS. Scale bar indicates 100 μm. (**b**) Epidermal thickness of FTMs generated under normoxia or hypoxia and NHS, indicated by mean + s.d. (**c**) Stratum corneum thickness of FTMs generated under normoxia or hypoxia and NHS. Data indicates mean + s.d. (**d**) HIF target gene expression in the epidermis of FTMs cultured under hypoxia as compared to normoxia, indicated by mean ± s.e.m., n = 4. Statistical differences are noted as *, ** or ***, corresponding to P < 0.05, <0.01, <0.001.

Next, evaluation of the effect of oxygen level in 3D co-cultures of fibroblasts and keratinocytes was performed by generation of full thickness models (FTMs) under normoxia or hypoxia. In both conditions, FTMs have a similar appearance based on macroscopic observations and the detection of four distinguishable epidermal layers (i.e. the stratum basale, stratum spinosum, stratum granulosum and SC) in hematoxylin and eosin (HE) stained cross sections (Fig. 2a). The epidermis remained well-ordered under hypoxia and the thickness of the viable epidermis was significantly reduced, better resembling that of NHS (Fig. 2b). The number of corneocyte layers in the SC of FTMs in hypoxia was significantly reduced as compared to normoxia and was similar to NHS (Fig. 2c). Likewise as in monocultures, upregulation of HIF target genes VEGFA, PDK1 and GLUT1 was observed in the epidermis of FTMs generated under hypoxia (Fig. 2d).

### Morphogenesis of FTMs generated under hypoxia

**Figure 3:**
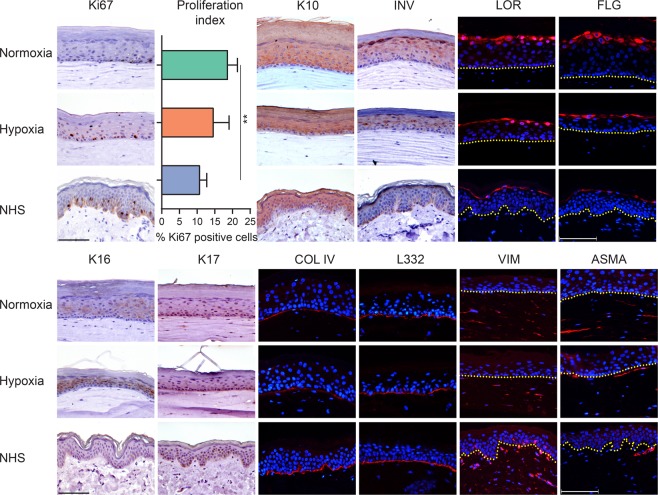
Morphogenesis of the epidermis and dermis in FTMs under hypoxia. Expression of protein biomarkers of proliferation (Ki67), early differentiation (K10), late and terminal differentiation (involucrin, loricrin, filaggrin), epidermal cell activation (K16 and K17), deposition of basement membrane (collagen type IV and laminin 332), fibroblasts distribution (vimentin) and fibroblasts stress signaling (alpha smooth muscle actin). Protein expression is shown in FTMs developed under normoxia or hypoxia and in NHS. Nuclei are counterstained blue using hematoxylin or DAPI, yellow dotted line indicates dermal-epidermal junction. Scale bar indicates 100 μm. Proliferation index is indicated by mean + s.d.

To obtain further information on the influence of oxygen levels on FTMs, the expression of specific biomarkers for dermal and epidermal morphogenesis was examined in the various FTMs generated under normoxia and hypoxia and these were compared to NHS (Fig. 3). The expression of the cell proliferation biomarker Ki67 remained restricted to the basal cell layer in all conditions. The proliferation was considerably higher in FTMs generated under normoxia as compared to NHS. However, the proliferation index of FTMs generated under hypoxia was lower than in normoxia, thereby mimicking that of NHS better. The early differentiation program was executed correctly in all conditions as indicated by suprabasal keratin 10 (K10) expression. The late and terminal differentiation programs were attenuated, as the keratinocytes in the granular layer appeared more flattened based on the expression of involucrin (INV), loricrin (LOR) and filaggrin (FLG). Epidermal cell activation was determined by expression of biomarkers K16 and K17. In FTMs generated in hypoxia, increased expression of K16 was observed as compared to normoxia, whereas K17 remained unexpressed. Both biomarkers were absent in NHS. Next, we determined the deposition of the basement membrane (BM) proteins collagen type IV (COL IV) and laminin 332 (L332). These were more continuously present in NHS and had a patchier similar appearance in both FTMs. The distribution of fibroblasts was studied by the expression of the mesenchymal biomarker vimentin (VIM). In FTMs generated under normoxia or hypoxia, the distribution was continuous and comparable, whereas in NHS there was a clear distinction between papillary and reticular dermis. Finally, the fibroblasts differentiation was determined by assessment of expressed alpha smooth muscle actin (ASMA), specific for myofibroblasts. No differences for this fibroblast characteristic were found in the FTMs generated under normoxia or hypoxia. In NHS, ASMA expression was also observed in dermal regions surrounding the capillaries, which were absent in FTMs (*data not shown*).

### Stratum corneum lipid composition of FTMs under hypoxia

**Figure 4:**
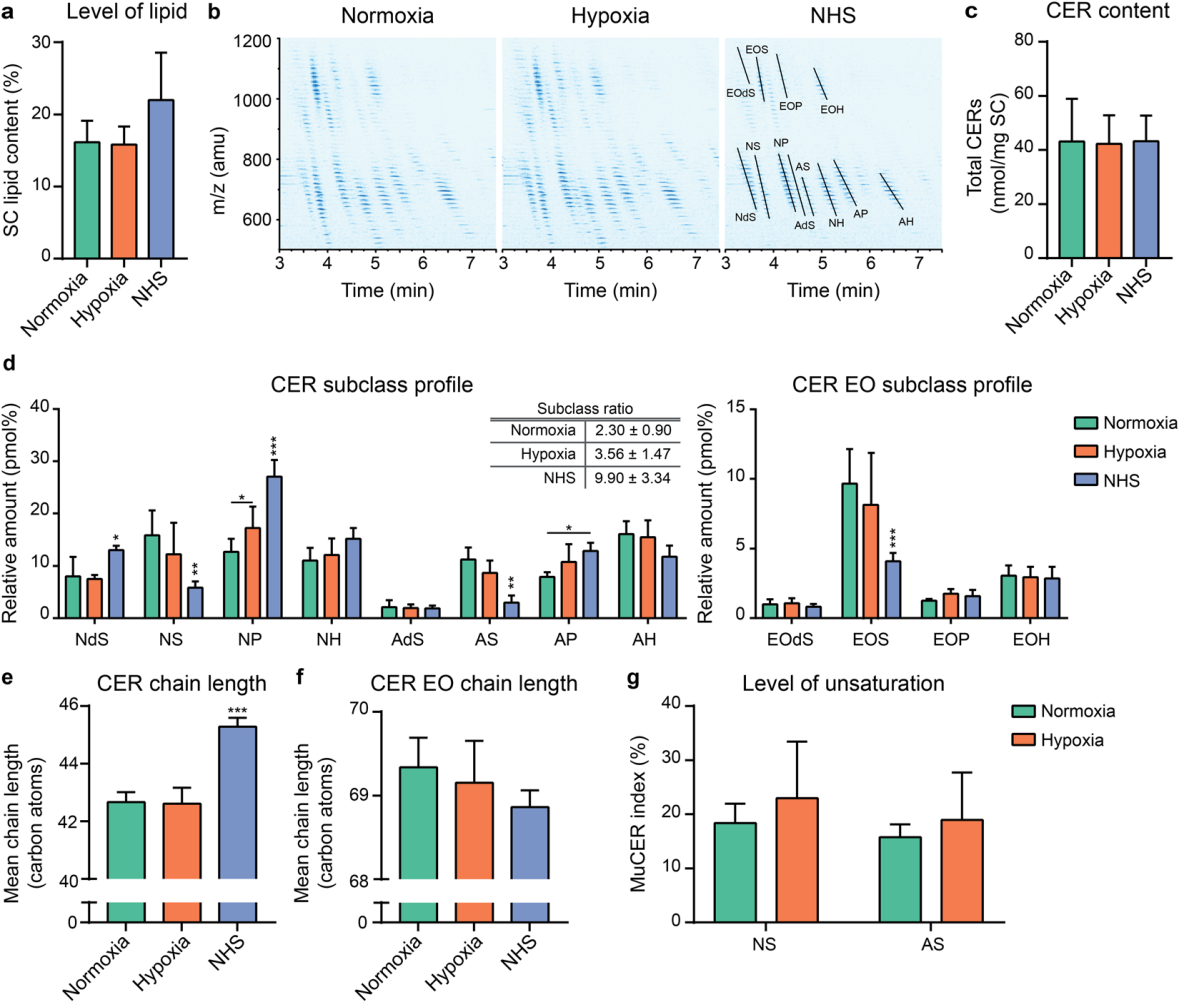
Stratum corneum ceramide composition of FTMs generated under hypoxia. (**a**) Level of total lipids in the SC of FTMs generated at indicated oxygen level and NHS. **(b)** Ion maps as detected by LC-MS of FTMs generated under normoxia or hypoxia and NHS. Twelve CERs_total_ subclasses are identified. **(c)** Cumulative amount of quantified CERs_total_ per mg SC in FTMs and NHS. **(d)** CER and CER EO subclass profile of FTMs and NHS. Tabular inset provides subclass ratio defined by $\frac{(dS+P+H)}{S}$, as mean ± s.d. **(e)** Bar diagram plot compares the CER mean chain length in FTMs and NHS. **(f)** Bar diagram plot compares the CER EO mean chain length in FTMs and NHS. **(g)** Level of unsaturation in CER subclasses NS and AS in FTMs. All data represents mean + s.d. Significance is shown for comparison between normoxia and hypoxia (with lines) and for comparison between FTMs and NHS (above NHS), otherwise indicated. Statistical differences are noted as *, ** or ***, corresponding to P < 0.05, <0.01, <0.001.

Thereafter, the lipid barrier formation was studied in FTMs generated under normoxia or hypoxia and compared to NHS. To this end, lipids were extracted from the SC, which revealed an equal total lipid content in the SC of FTMs in both conditions and NHS (Fig. 4a). Next, we determined the CERs_total_ composition within the lipid extract using a liquid chromatography coupled to mass spectrometry analysis. In FTMs generated under normoxia and hypoxia, all twelve major CER subclasses were detected (Fig. 4b). As compared to the ion map of NHS, in that of FTMs there is an increased presence of CERs with a mass below 600, indicative for increased presence of CERs with a shorter carbon chain length. Quantification of the CERs_total_ in absolute molar values revealed a similar cumulative amount of CERs_total_ per mg of SC in FTMs and NHS (Fig. 4c). Subsequently, the CER and CER EO subclass profiles were analyzed (Fig. 4d). An altered CER subclass profile in hypoxic FTMs was detected with significant higher abundance of subclass NP. This also improved the CER subclass ratio defined by $\frac{(dS+P+H)}{S}$, which mimics more closely that of NHS. The CER EO subclass profile of the FTMs did not differ, although the composition of CERs EO in FTMs deviated from that in NHS. Next, the mean chain length (MCL) of the CERs and CERs EO was determined (Fig. 4e,f). High similarity was observed between the FTMs for the MCL of the CERs and of the CERs EO. As compared to that of NHS, there is a significant reduction observed in the MCL of the CERs, but not in that of the CERs EO. In line with the MCL, no differences in the carbon chain length distribution of the CERs_total_ in the FTMs generated at both conditions were detected (Supplementary Fig. 3). When comparing the carbon chain length distribution of FTMs to that of NHS, the main difference is the increased presence of CERs with ≤42 carbon atoms and the reduced presence of CERs with ≥44 carbon atoms (Supplementary Fig. 3a). No major differences were observed in the carbon chain length distribution of CERs EO of FTMs, although substantially altered as compared to that of NHS (Supplementary Fig. 3b). Hereafter, the level of unsaturation in the CER subclasses AS and NS was determined. These subclasses were selected, as changes in the level of unsaturation in these subclasses properly indicate differences in the level of unsaturation for CERs_total_ (Helder *et al*., in preparation). The unsaturation index indicates the percentage of monounsaturated CERs per subclasses. When comparing the unsaturation index between FTMs developed under normoxia to hypoxia, a similar unsaturation index was observed (Fig. 4g). As monounsaturation in ceramides is naturally low in NHS, this was not further analyzed.

### Stratum corneum lipid organization of FTMs under hypoxia

**Figure 5:**
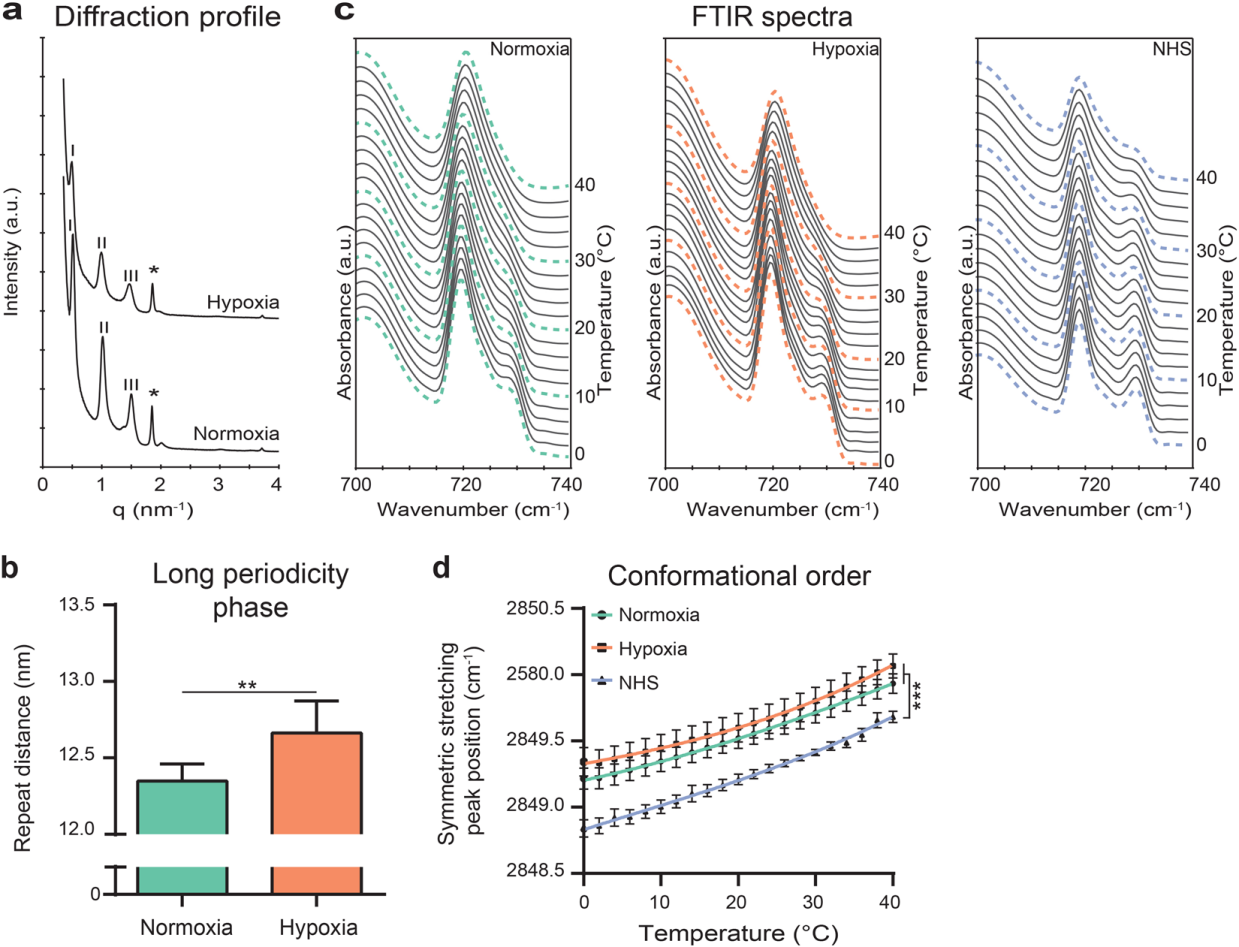
Lipid organization in FTMs generated under hypoxia. (**a**) Small angle X-ray diffraction profiles of FTMs developed under normoxia and hypoxia. Orders of diffraction of the LPP are indicated by roman numbers and phase separated crystalline cholesterol is indicated by the asterisk (*). **(b)** Bar diagram plot of the long periodicity phase repeat distances in both FTMs. Data represent mean + s.d. Statistical testing is performed by paired t-test. **(c)** Representative rocking vibrations in a temperature range of 0–40 °C in FTMs developed under normoxia, hypoxia and of NHS. **(d)** Conformational ordering of the lipids based on the symmetric stretching peak position over a temperature range of 0–40 °C. Third order polynomial regression line connects the data points. Data represents mean ± s.e.m., n = 4.

After assessment of the ceramide composition, the organization of the lipids in the intercorneocyte space was determined in FTMs generated under normoxia or hypoxia and compared to NHS. The lamellar organization was examined by small-angle X-ray diffraction. The diffraction profile with a first, second, and third order of diffraction attributed to the long periodicity phase (LPP) were detected in FTMs, irrespective of oxygen level (Fig. 5a). From the positions of the diffraction peaks, the repeat distance of the LPP in both FTMs was calculated. This revealed an increase in the repeat distance of the LPP in FTMs generated under hypoxia (Fig. 5b). In NHS, by using recrystallization studies, the LPP repeat distance has been reported before to be around 13 nm^34^. Therefore, the LPP repeat distance in the FTMs generated in hypoxia better resembles the repeat distance in NHS. The lateral packing of FTMs generated under normoxia or hypoxia and NHS was evaluated by Fourier transformed infrared spectrometry (FTIR) measurements. Examination of the methylene rocking vibration in the 700–740 cm^−1^ region of the spectrum provides information about the lipid lateral packing; two peaks at 720 and 730 cm^−1^ indicate lipids assembled in the very dense orthorhombic packing, whereas a single peak at 720 cm^−1^ is visible when the lipids form a hexagonal packing^15^. In FTMs, a weak doublet shoulder was visible at low temperatures (Fig. 5c). This indicated that a small fraction of the lipids assembled in an orthorhombic lateral packing, but that most lipids form a hexagonal lateral packing. When increasing the temperature, the intensity of the 730 cm^−1^ shoulder gradually reduced. At 40 °C, the lipids assembled only in a hexagonal packing, which was similar for normoxia and hypoxia. In NHS, the 730 cm^−1^ peak intensity was higher at low temperatures. This indicated that a higher fraction of lipids was organized in the orthorhombic lateral packing in NHS. When increasing the temperature, the intensity of the 730 cm^−1^ peak reduced, but a doublet was still present at 40 °C, indicating a substantial level of lipids still adapted an orthorhombic packing. The conformational ordering of the lipids was examined to determine the ordered-disordered phase behavior of the lipid matrix. This could be obtained by assessment of the methylene symmetric stretching vibration peak position in the FTIR spectrum. When lipid chains adapt a higher conformational ordering, these are fully extended. This is indicated by methylene stretching vibration peaks positioned below a wavenumber of 2850 cm^−1^. Vibration peaks shifted to a higher wavenumber (2852 cm^−1^) when higher disordering of lipids is present. Peak positions of methylene symmetric stretching vibrations of FTMs and of NHS are plotted against temperature range of 0–40 °C (Fig. 5d). The conformational ordering of the lipids in FTMs generated at normoxia or hypoxia was comparable. In NHS, lowest vibrations were detected, indicative for highest conformational ordering of the lipid tails. In FTM and NHS, the lipids adopt a crystalline phase, as the observed frequency is below 2850 cm^−1^ at skin surface temperature of 32 °C.

## Discussion

The main objective of this study is to investigate the influence of oxygen level on dermal and epidermal morphogenesis and barrier formation in HSEs. Our results demonstrate that a reduced oxygen level (i) activates HIF signaling *in vitro* leading to metabolic reprogramming of primary human fibroblasts and keratinocytes in mono and 3D cultures, (ii) decreases epidermal thickness in FTMs, (iii) affects epidermal morphogenesis, and (iv) alters the SC lipid composition and organization. These alterations led to better resemblance of HSEs to NHS.

The results obtained in this study are in line with previous studies, which demonstrated an altered expression of several barrier proteins including FLG, LOR and INV under hypoxia, indicative for a restructured barrier formation^33,35^. Furthermore, silencing of HIF-α affected the terminal differentiation program in a human epidermal model that resulted in impaired stratification of the epidermis^32^. A similar observation was made *in vivo*, where it has been shown that the lack of HIF-α results in an impaired terminal differentiation program and barrier formation in a murine model^33^. This emphasizes the importance of oxygen levels and HIF signaling in the formation of the SC barrier. An abnormal differentiation under hypoxia has also been reported, as determined by a reduced K1/K10 and FLG/LOR expression in HSEs after 24 hours at 1% oxygen^36^. However, the level of ROS scavenger molecules (vitamin C and E) and the duration and level of oxygen reduction (1% vs 3%) differ between this and our study, in part explaining the divergent outcomes. Complementary to our results, hyperbaric oxygen treatment (hyperoxia) of HSEs accelerated the formation of the epidermis, concomitantly with an increased proliferation rate and enlargement of the granular cell layer, which again emphasizes the critical role of oxygen during skin tissue engineering^37,38^.

The lipid barrier formation is enhanced in FTMs generated under hypoxia, as it mimics that of NHS more closely than in FTMs generated under normoxia. Of interest is the significant improvement in CER subclass NP level and improved subclass ratio $\frac{(dS+P+H)}{S}$ under hypoxia. An increase in this subclass or in the subclass ratio is also one of the key alterations observed *in vivo* in diseased skin conditions^39,40^ and is shown to be directly correlated with the skin barrier function^41^. Lipid elongation or the level of unsaturation in subclasses AS and NS is not affected by culturing under hypoxia, indicating that other regulatory pathways are involved in these processes. In this study, we thoroughly characterized the barrier formation in HSEs by full quantification of the ceramide composition. Due to the technical complexity of the data analysis, no unsaturated CERs EO with a linoleic chain (contributing ~17–19% to FTM and NHS, *Helder et al., in preparation*) and saturated CERs EO with an oleic chain (contributing ~23% to FTM, *Helder et al., in preparation*) were analyzed. However, the interpretation of the data is not affected by this. The ceramide composition affects the lipid organization as shown in this study and others^40,42^, but also for the barrier functionality, as demonstrated in *in vivo* (diseased) skin conditions^40,43^, in HSEs^9,42^ and in pure lipid model membranes^44,45^. The influence of reduced oxygen level on the presence of other major lipid classes (free fatty acids, cholesterol) could be of interest in future studies.

Incorporation of the optimized oxygen micro-environment in future projects could open new possibilities to study different aspects of skin biology. One of these is a 3D co-culture with vascular endothelial cells. These are often supplemented with high concentrations of synthetic VEGF^46–48^, whereas under hypoxia the production of endogenous VEGF is upregulated, potentially leading to a more supportive micro-environment. This facilitates studies on dermal angiogenesis, which is an important aspect in wound healing and tumorigenesis^49,50^. Furthermore, a low oxygen micro-environment could also be applied in human skin organ culture (hSOC)^51^. This enables the determination of the effect of hypoxia on other skin resident cell populations, which are currently missing in FTMs. Additionally, the interplay between oxygen levels in the skin micro-environment with the ability of the epidermis to induce anti-microbial responses during bacterial (*S. aureus*) infections was reported recently^52,53^. Essentially, hypoxia lowers the threshold for induction of these protective responses, suggesting a role of oxygen level during the dynamic anti-microbial barrier^54^, besides its role on the structural barrier formation.

In summary, we have shown in monocultures and in FTMs that external oxygen level directly affects cellular proliferation rate, epidermal morphogenesis and barrier formation. Importantly, we have shown that sub-optimized external conditions are in part responsible for altered *in vitro* skin tissue reconstruction.

## Materials and Methods

### Isolation and culturing of primary fibroblasts and keratinocytes

Declaration of Helsinki principles are followed during obtainment of primary cells from human skin tissue originating from surplus breast tissue of healthy donors. Experiments were conducted in accordance with article 7:467 of the Dutch Law on Medical Treatment Agreement and the Code for proper Use of Human Tissue of the Dutch Federation of Biomedical Scientific Societies (https://www.federa.org/codes-conduct). As of this national legislation, coded surplus tissue can be used for scientific research purposes when no written objection in made by the informed donor, i.e. an informed opt-out system. Therefore, additional approval of an ethics committee regarding scientific use of surplus tissue was neither requested nor required, as stated earlier^55^. Primary cells were isolated from surplus human mamma skin after separation of the epidermis and dermis by incubation in 2.4 U/mL dispase II (Roche, Almere, The Netherlands). Isolation and culturing of primary cell suspensions was performed as described before^42,56,57^. All isolated primary cells were tested and found negative for mycoplasma contamination. Amount of viable proliferating primary cells was determined by trypan blue exclusion assay using a Bio-Rad TC20™ automatic cell counter (Bio-Rad, Hercules, CA, USA) according to manufacturer’s instructions.

### Generation of full thickness models

Full thickness models (FTMs) were generated in two steps and cultured on inert membranes using a transwell system (Corning Transwell cell culture inserts, membrane diameter 24 mm, pore size 3 μm; Corning Life Sciences, The Netherlands) as described before^9,42^. In the first step, the dermal equivalents were generated using primary fibroblasts (1.2–1.5 × 10^5^) in passage number 2–5, hydrated rat-tail tendon collagen (4 mg/mL), 1 M NaOH, Hank’s Balanced Salt Solution, and 5% fetal bovine serum (FBS; GE Healthcare, Chicago, IL, USA). After polymerization at 37 °C, the dermal equivalents were cultured 7 days as described elsewhere^9,42^. In the second step, the epidermal equivalents were generated by seeding primary keratinocytes (2.5 × 10^5^/model) in passage number 1 directly onto each dermal equivalent as reported before^9,57^. The FTMs were kept submerged for total of four days. Hereafter, the FTMs were lifted to the air-liquid interface and cultured for 14–15 days under normoxia (20%) in the Memmert INC153med CO_2_ incubator (Memmert, Schwabach, Germany) or under hypoxia (3%) in the Heracell™ 240 CO_2_ incubator (ThermoFisher, Waltham, MA, USA). FTM batches were generated with 5 different primary cell donors.

### Gene expression

#### RNA isolation

Isolation of RNA from 2D and 3D cell cultures was performed according to instructions provided by the manufacturer using a FavorPrep Tissue Total RNA Mini Kit (Favorgen, Ping-Tung, Taiwan). A single exception was the incorporation of a DNA digestion step, which occured during 15 minutes after the initial washing steps using an RNAse-free DNAse solution (Qiagen, Hilden, Germany). Subsequently, the isolated RNA was eluted and the concentration of the nucleic acid content was determined using a NanoDrop™ UV-Vis Spectrophotometer (ThermoFisher).

#### Quantitative real-time polymerase chain reaction

A total of 500 ng isolated RNA was used to synthesize complementary DNA with an iScript™ cDNA Synthesis Kit (Bio-Rad). Then, quantitative real-time polymerase chain reactions were performed with the SYBR Green Supermix (Bio-Rad) using the CFX384 Touch™ real-time PCR detection system (Bio-Rad). Used settings were described previously by Mieremet *et al*.^58^. Details on primer sequences used in this study are described in Supplementary Table 1.

### Tissue morphology

#### Tissue fixation

Sections of FTMs or NHS were fixated with formaldehyde for paraffin embedding or snap frozen for cryofixation. For paraffin embedding, the tissue was fixated overnight in 4% (w/v) formaldehyde (Added Pharma, Oss, The Netherlands), dehydrated and embedded in paraffin. For cryofixation, the tissue was placed in a gelatin capsule containing Tissue-Tek® O.C.T.™ Compound (Sakura Finetek Europe, Alphen aan den Rijn, The Netherlands), snap frozen in liquid nitrogen and stored at −80 °C.

#### Protein expression

General morphological assessments were performed on 5 μm paraffin embedded tissue sections after staining with hematoxylin and eosin (HE; Klinipath, Duiven, The Netherlands). Immunohistochemical and immunofluorescence analyses of protein expression were performed on 5 μm sections. FFPE material was deparaffinized and rehydration, whereas frozen material was dried overnight and fixed in acetone for 10 minutes. For FFPE material, heat-mediated antigen retrieval in citrate buffer was performed, followed by blocking with normal human serum (Sanquin, Leiden, The Netherlands). Stainings were performed using the streptavidin-biotin-peroxidase system (GE Healthcare) according to the manufacturer’s instructions or using indirect immunofluorescence, as described elsewhere^42,59^. Exceptions were the ASMA and collagen type IV staining, for which no or protease-mediated antigen retrieval was performed respectively^42^. Visualization of the sections occurred using a light microscope (Zeiss Axioplan 2, Carl Zeiss BV, Breda, The Netherlands) or a fluorescence microscope (Leica CTR5000, Leica, Wetzlar, Germany). Application of solely secondary antibodies revealed no interfering background or unspecific staining (Supplementary Fig. 4a). Specifications of the materials are provided in Supplementary Table 2.

#### Determination of the number of corneocyte layers

Snap-frozen samples were cut 5 μm thickness using a Leica CM3050S cryostat (Leica Biosystems, Nussloch, Germany). Sections were placed on a Superfrost® Plus Micro Slide (VWR, Radnor, USA), air-dried overnight, and fixated in ice-cold acetone for a minimum of 10 minutes. Samples were stained with a 1% (w/v) safranin O (Sigma-Aldrich, St. Louis, MO, USA) solution for 1 minute. Subsequently, samples were immersed with a 2% (w/v) KOH solution for 10–15 minutes, which induced swelling of the corneocytes. Samples were covered and imaged directly after the procedure to determine the number of corneocyte layers (Supplementary Fig. 4b).

#### Estimation of epidermal thickness and proliferation index

Procedures to estimate the epidermal thickness and the proliferation index were described before^60^. In short, epidermal thickness was determined by digital quantification of the viable epidermis in the HE stained samples. Proliferation index was determined by counting the number of Ki67 positive nuclei in a region of the basal epidermal layer covering at least 100 keratinocytes. A minimum of three different epidermal regions were included.

### Stratum corneum lipid composition

#### Stratum corneum isolation

Isolation of the stratum corneum occurred after overnight incubation in a 0.1% (w/v) trypsin/PBS solution at 4 °C followed by incubation at 37 °C for 1 hour. The SC was peeled off the epidermis and was washed with 0.1% trypsin inhibitor (Sigma) and Millipore water. The isolates were air-dried and stored at dry conditions.

#### Lipid extraction and liquid chromatography – mass spectrometry

Extraction of barrier lipids from the SC was carried out using a modified Bligh and Dyer procedure^61^ as described by Boiten *et al*.^62^. To determine the amount of material extracted from the SC, the SC was dried and weighed before as well as after the extraction procedure. The obtained lipid extract was dissolved in a suitable volume of heptane:chloroform:methanol (95:2.5:2.5 v:v:v). The lipids were analyzed and quantified using normal phase liquid chromatography - mass spectrometry (LC-MS) analysis, as described by Boiten *et al*.^62^. In brief, SC lipid extracts were separated on a PVA-Sil column (5 μm particles, 100 × 2.1 mm ID; YMC, Kyoto, Japan) with an Acquity UPLC H-class (Waters, Milford, MA, USA). CERs_total_ were detected by an XEVO TQ-S mass spectrometer (Waters). LC-MS measurements occurred in full scan mode with settings for time between 1.25–8.00 minutes at m/z 350–1350 and for time between 8.0–12.5 minutes at m/z 500–1350.

#### Data quantification

The area under the curve (AUC) of a chromatogram from the mono-isotopic mass of the main fragment was integrated. For a selected number of CERs, the relative abundance of the corresponding unsaturated CER containing two ^13^C was more than 10%. When this occurred, the AUC of the two ^13^C containing CER was calculated using its natural isotope distribution. This AUC was subtracted from the one of with which it overlapped. Quantification of the molar amount of ceramides occurred using the AUC of the chromatogram of any specific ceramide, the internal standard (INST) of CER N(24deuterated)S(18) and a three dimensional response model based on compound properties and a calibration curve from a limited number of synthetic ceramides according to the method described by Boiten *et al*.^62^ (Supplementary Fig. 5). Nomenclature of the CER subclasses is followed according to Motta *et al*.^63^ and is shown in Supplementary Fig. 2.

### Stratum corneum lipid organization

#### Small-angle X-ray diffraction

Small-angle x-ray diffraction analyses occurred at station BM26B of the European Synchrotron Radiation Facility using methods specified before by Mojumdar *et al*.^64^. The detector was calibrated with silver behenate and cholesterol. Sample measurements occurred twice for 90 seconds at two detector positions. Distance of the sample to the pilatus1M detector was approximately 2.1 meter. Profiles were obtained by converting the detector image from Cartesian (x, y) to polar (r, θ) coordinates by integrating over scattering angle θ. The scattering intensity was determined as a function of the scattering vector q, which was defined as $q=\frac{4\cdot \pi \cdot \,\sin \,\theta }{\lambda }$, in which λ indicates the wavelength. Based on the positions of the peaks, the repeat distance was calculated according to the equation $d=\frac{2n\cdot \pi }{qn}$, where n indicates the order of the diffraction peak. Determination of the repeat distance of each lamellar phase was performed using combined data from the three main diffraction peaks.

#### Fourier transform infrared spectrometry

Fourier transform infrared spectroscopy (FTIR) spectra were obtained using a Varian 670-IR spectrometer (Agilent Technologies, Santa Clara, CA, USA). The system was equipped with a broad-band mercury cadmium telluride detector, cooled with liquid nitrogen, and connected to an external controlled heating device. Before measurements, the SC was sandwiched between two AgBr cells in parallel to their surface. A constant stream of dry air was applied for 30 minutes to prevent signal interference by water condensations. The FTIR settings are described by Mieremet *et al*.^58^. Spectra were analyzed and deconvoluted using Varian Resolutions Pro 5.2.0 software (Agilent Technologies).

### Statistics

Statistical examinations were performed with GraphPad Prism v7.00 (GraphPad Software, La Jolla, CA, USA). In general, statistical testing was performed with 1-way or 2-way ANOVA with Tukey’s multiple comparisons post-test, otherwise indicated. Significance is shown for comparison between normoxia and hypoxia (with lines) and for comparison between FTMs and NHS (above NHS), otherwise indicated. Significant differences were indicated by *, ** or ***, which correspond to P values < 0.05, <0.01, <0.001, respectively.

## Supplementary information

SREP-18-27962B Supplementary Information

## Data Availability

The raw data obtained during this study and processed data used for analysis is available upon reasonable request from the senior authors.

## References

[1] Gordon S (2015). Non-animal models of epithelial barriers (skin, intestine and lung) in research, industrial applications and regulatory toxicology. Alternatives to Animal Experimentation: ALTEX.

[2] Sun T, Jackson S, Haycock JW, MacNeil S (2006). Culture of skin cells in 3D rather than 2D improves their ability to survive exposure to cytotoxic agents. Journal of biotechnology.

[3] Helmedag MJ (2014). The effects of constant flow bioreactor cultivation and keratinocyte seeding densities on prevascularized organotypic skin grafts based on a fibrin scaffold. Tissue Engineering Part A.

[4] van Drongelen V, Haisma EM, Out‐Luiting JJ, Nibbering P, El Ghalbzouri A (2014). Reduced filaggrin expression is accompanied by increased Staphylococcus aureus colonization of epidermal skin models. Clinical & Experimental Allergy.

[5] Mathes SH, Ruffner H, Graf-Hausner U (2014). The use of skin models in drug development. Advanced drug delivery reviews.

[6] van den Broek LJ, Bergers LI, Reijnders CM, Gibbs S (2017). Progress and future prospectives in skin-on-chip development with emphasis on the use of different cell types and technical challenges. Stem Cell Reviews and Reports.

[7] Sriram G (2018). Full-thickness human skin-on-chip with enhanced epidermal morphogenesis and barrier function. Materials Today.

[8] Niehues H (2018). 3D skin models for 3R research: the potential of 3D reconstructed skin models to study skin barrier function. Experimental dermatology.

[9] Thakoersing VS (2011). Unraveling barrier properties of three different in-house human skin equivalents. Tissue Engineering Part C: Methods.

[10] Schreiber S (2005). Reconstructed epidermis versus human and animal skin in skin absorption studies. Toxicology in vitro.

[11] Schmook FP, Meingassner JG, Billich A (2001). Comparison of human skin or epidermis models with human and animal skin in *in-vitro* percutaneous absorption. International journal of pharmaceutics.

[12] Maxwell G (2014). Applying the skin sensitisation adverse outcome pathway (AOP) to quantitative risk assessment. Toxicology in Vitro.

[13] Brandner J (2015). Epidermal tight junctions in health and disease. Tissue barriers.

[14] Yokouchi M (2016). Epidermal cell turnover across tight junctions based on Kelvin’s tetrakaidecahedron cell shape. Elife.

[15] van Smeden J, Janssens M, Gooris GS, Bouwstra JA (2014). The important role of stratum corneum lipids for the cutaneous barrier function. Biochimica et Biophysica Acta (BBA) - Molecular and Cell Biology of Lipids.

[16] Feingold KR, Elias PM (2014). Role of lipids in the formation and maintenance of the cutaneous permeability barrier. Biochimica et Biophysica Acta (BBA)-Molecular and Cell Biology of Lipids.

[17] Vávrová K, Kováčik A, Opálka L (2017). Ceramides in the skin barrier. European Pharmaceutical Journal.

[18] Kihara A (2016). Synthesis and degradation pathways, functions, and pathology of ceramides and epidermal acylceramides. Progress in lipid research.

[19] van Smeden J, Bouwstra JA (2016). Stratum corneum lipids: their role for the skin barrier function in healthy subjects and atopic dermatitis patients. Current Problems in Dermatology.

[20] Harayama T, Riezman H (2018). Understanding the diversity of membrane lipid composition. Nature Reviews Molecular Cell Biology.

[21] Rabionet M, Gorgas K, Sandhoff R (2014). Ceramide synthesis in the epidermis. Biochimica et Biophysica Acta (BBA)-Molecular and Cell Biology of Lipids.

[22] Thakoersing VS (2013). Increased presence of monounsaturated fatty acids in the stratum corneum of human skin equivalents. Journal of Investigative Dermatology.

[23] Tfayli A (2014). Comparison of structure and organization of cutaneous lipids in a reconstructed skin model and human skin: spectroscopic imaging and chromatographic profiling. Experimental dermatology.

[24] Moore TC, Hartkamp R, Iacovella CR, Bunge AL, McAbe C (2018). Effect of Ceramide Tail Length on the Structure of Model Stratum Corneum Lipid Bilayers. Biophysical Journal.

[25] Evans SM, Schrlau AE, Chalian AA, Zhang P, Koch CJ (2006). Oxygen Levels in Normal and Previously Irradiated Human Skin as Assessed by EF5 Binding. Journal of Investigative Dermatology.

[26] Rezvani HR (2011). HIF-1α in Epidermis: Oxygen Sensing, Cutaneous Angiogenesis, Cancer, and Non-Cancer Disorders. Journal of Investigative Dermatology.

[27] Place TL, Domann FE, Case AJ (2017). Limitations of oxygen delivery to cells in culture: An underappreciated problem in basic and translational research. Free Radical Biology and Medicine.

[28] Weidemann A, Johnson R (2008). Biology of HIF-1α. Cell Death & Differentiation.

[29] Wu D, Potluri N, Lu J, Kim Y, Rastinejad F (2015). Structural integration in hypoxia-inducible factors. Nature.

[30] Seleit I, Bakry OA, Al-Sharaky DR, Ragab RAA (2017). Evaluation of Hypoxia Inducible Factor-1α and Glucose Transporter-1 Expression in Non Melanoma Skin Cancer: An Immunohistochemical Study. Journal of Clinical and Diagnostic Research.

[31] Maxwell PH (1999). The tumour suppressor protein VHL targets hypoxia-inducible factors for oxygen-dependent proteolysis. Nature.

[32] Rezvani HR (2011). Loss of epidermal hypoxia-inducible factor-1α accelerates epidermal aging and affects re-epithelialization in human and mouse. Journal of Cell Science.

[33] Wong WJ, Richardson T, Seykora JT, Cotsarelis G, Simon MC (2015). Hypoxia-inducible factors regulate filaggrin expression and epidermal barrier function. Journal of Investigative Dermatology.

[34] Bouwstra JA, Gooris GS, van der Spek JA, Bras W (1991). Structural investigations of human stratum corneum by small-angle X-ray scattering. Journal of investigative dermatology.

[35] Li H, Zhou L, Dai J (2018). Retinoic acid receptor‐related orphan receptor RORα regulates differentiation and survival of keratinocytes during hypoxia. Journal of cellular physiology.

[36] Park J-y (2016). Hypoxia leads to abnormal epidermal differentiation via HIF-independent pathways. Biochemical and biophysical research communications.

[37] Kairuz E, Upton Z, Dawson RA, Malda J (2007). Hyperbaric oxygen stimulates epidermal reconstruction in human skin equivalents. Wound Repair and Regeneration.

[38] Malda J, Klein TJ, Upton Z (2007). The roles of hypoxia in the *in vitro* engineering of tissues. Tissue engineering.

[39] Farwanah H, Raith K, Neubert RH, Wohlrab J (2005). Ceramide profiles of the uninvolved skin in atopic dermatitis and psoriasis are comparable to those of healthy skin. Archives of dermatological research.

[40] Janssens M (2012). Increase in short-chain ceramides correlates with an altered lipid organization and decreased barrier function in atopic eczema patients. Journal of lipid research.

[41] Boiten WA (2018). Applying a vernix caseosa based formulation accelerates skin barrier repair by modulating lipid biosynthesis. Journal of lipid research.

[42] Mieremet A (2017). Improved epidermal barrier formation in human skin models by chitosan modulated dermal matrices. PloS one.

[43] Coderch L, López O, de la Maza A, Parra JL (2003). Ceramides and Skin Function. American Journal of Clinical Dermatology.

[44] Školová B, Kováčik A, Tesař O, Opálka L, Vávrová K (2017). Phytosphingosine, sphingosine and dihydrosphingosine ceramides in model skin lipid membranes: permeability and biophysics. Biochimica et Biophysica Acta (BBA) - Biomembranes.

[45] Mojumdar E, Kariman Z, van Kerckhove L, Gooris G, Bouwstra J (2014). The role of ceramide chain length distribution on the barrier properties of the skin lipid membranes. Biochimica et Biophysica Acta (BBA)-Biomembranes.

[46] Ponec M (2004). Endothelial network formed with human dermal microvascular endothelial cells in autologous multicellular skin substitutes. Angiogenesis.

[47] Auxenfans C (2012). Adipose‐derived stem cells (ASCs) as a source of endothelial cells in the reconstruction of endothelialized skin equivalents. Journal of tissue engineering and regenerative medicine.

[48] Monsuur HN (2016). Extensive Characterization and Comparison of Endothelial Cells Derived from Dermis and Adipose Tissue: Potential Use in Tissue Engineering. Plos One.

[49] Chuan T (2016). Hypoxia pretreatment of bone marrow—derived mesenchymal stem cells seeded in a collagen‐chitosan sponge scaffold promotes skin wound healing in diabetic rats with hindlimb ischemia. Wound Repair and Regeneration.

[50] Kur-Piotrowska A (2018). Foxn1 expression in keratinocytes is stimulated by hypoxia: further evidence of its role in skin wound healing. Scientific reports.

[51] Zhou L, Zhang X, Paus R, Lu Z (2018). The renaissance of human skin organ culture: A critical reappraisal. Differentiation.

[52] Peyssonnaux C (2008). Critical Role of HIF-1α in Keratinocyte Defense against Bacterial Infection. Journal of Investigative Dermatology.

[53] Wickersham M (2017). Metabolic stress drives keratinocyte defenses against staphylococcus aureus infection. Cell reports.

[54] Rademacher F, Simanski M, Gläser R, Harder J (2018). Skin microbiota and human 3D skin models. Experimental dermatology.

[55] Haisma EM (2013). Inflammatory and antimicrobial responses to methicillin-resistant Staphylococcus aureus in an *in vitro* wound infection model. PloS one.

[56] El Ghalbzouri A, Commandeur S, Rietveld MH, Mulder AA, Willemze R (2009). Replacement of animal-derived collagen matrix by human fibroblast-derived dermal matrix for human skin equivalent products. Biomaterials.

[57] van Drongelen V (2014). Barrier properties of an N/TERT-based human skin equivalent. Tissue Engineering Part A.

[58] Mieremet A, van Dijk R, Gooris G, Bouwstra JA, El Ghalbzouri A (2019). Shedding light on the effects of 1,25-dihydroxyvitamin D_3_ on epidermal lipid barrier formation in three-dimensional human skin equivalents. Journal of Steroid Biochemistry and Molecular Biology.

[59] Mieremet A, Rietveld M, van Dijk R, Bouwstra JA, El Ghalbzouri A (2017). Recapitulation of Native Dermal Tissue in a Full-Thickness Human Skin Model Using Human Collagens. Tissue Engineering Part A.

[60] Mieremet, A. *et al*. Characterization of human skin equivalents developed at body’s core and surface temperatures. *Journal of Tissue Engineering and Regenerative Medicine (in press)*, 10.1002/term.2858 (2019). 10.1002/term.2858 PMC6767576 30945465

[61] Bligh EG, Dyer WJ (1959). A rapid method of total lipid extraction and purification. Canadian journal of biochemistry and physiology.

[62] Boiten W, Absalah S, Vreeken R, Bouwstra J, van Smeden J (2016). Quantitative analysis of ceramides using a novel lipidomics approach with three dimensional response modelling. Biochimica et Biophysica Acta (BBA)-Molecular and Cell Biology of Lipids.

[63] Motta S (1993). Ceramide composition of the psoriatic scale. Biochimica et Biophysica Acta (BBA)-Molecular Basis of Disease.

[64] Mojumdar EH, Helder RWJ, Gooris GS, Bouwstra JA (2014). Monounsaturated Fatty Acids Reduce the Barrier of Stratum Corneum Lipid Membranes by Enhancing the Formation of a Hexagonal Lateral Packing. Langmuir.

