# Supplementary material for: Human skin equivalents cultured under hypoxia display enhanced epidermal morphogenesis and lipid barrier formation
Source: Sci Rep. 2019 May 24;9:7811. doi: 10.1038/s41598-019-44204-4 (PMC6534609; doi:10.1038/s41598-019-44204-4)
Supplement: Supplementary file 1 — SREP-18-27962B Supplementary Information [file 41598_2019_44204_MOESM1_ESM.docx]

# Supplementary Information

**Article title:**

**Human skin equivalents cultured under hypoxia display enhanced epidermal morphogenesis and lipid barrier formation**

Author list:

Arnout Mieremet, Adela Vázquez García, Walter Boiten, Rianne van Dijk, Gert Gooris, Joke A. Bouwstra, and Abdoelwaheb El Ghalbzouri.

Supplementary table 1. Specifications of primer used for quantitative real-time polymerase chain reaction

| Target | Sequence Forward (5’-> 3’) | Sequence Reverse (5’-> 3’) | Amplicon size | Exonic location | Accession No. |
| --- | --- | --- | --- | --- | --- |
| SDHA | AACCAAACGCTGGGGAAGAA | GGAACACGGCAGCATGATTT | 126 | 11,12 | NM_004168.3 |
| ZNF410 | GCTGTGGTAAGCAGTTTACTACAG | CTTGGGCTTCACAAAGGAAAGG | 90 | 6,7 | NM_001242924.1 |
| ARCP2 | TCCGGGACTACCTGCACTAC | GGTTCAGCACCTTGAGGAAG | 96 | 9,10 | NM_152862.2 |
| VEGFA | ACTGAGGAGTCCAACATCACC | CTGCATTCACATTTGTTGTGCTG | 104 | 3,4 | NM_001025366.2 |
| PDK1 | CACCAGGACAGCCAATACAA | AACACCTCTGTTGGCATGGT | 116 | 7,8 | NM_002610.4 |
| GLUT1 | GGCGGGTTGTGCCATACTCA | CAAAGAAGGCCACAAAGCCAA | 107 | 8,9 | NM_006516.2 |

Supplementary table 2. Specification of antibodies used for immunohistochemical and immunofluorescence staining.

| **Immunohistochemistry** | **Material** | **Origin** | **Clone** | **Dilution** | **2^nd^ Ab** | **Manufacturer** |
| --- | --- | --- | --- | --- | --- | --- |
| ***Primary antibody*** | | | | | | |
| Ki67 | FFPE | Mouse | MIB1 | 1:100 | A | DAKO, Denmark |
| Cytokeratin 10 | FFPE | Mouse | DE-K10 | 1:50 | A | Labvision/Neomarker, USA |
| Involucrin | FFPE | Mouse | SY5 | 1:1200 | A | Sanbio, The Netherlands |
| Cytokeratin 16 | FFPE | Mouse | LL025 | 1:100 | A | Serotec, UK |
| Cytokeratin 17 | FFPE | Mouse | E3 | 1:1500 | A | EMD Millipore Corporation, USA |
| ***Secondary antibody*** | | | | | | |
| A) Biotinylated goat  anti-mouse |  | Goat |  | 1:200 |  | Southern Biotechnology, USA |
| **Immunofluorescence** |  | **Origin** | **Clone** | **Dilution** | **2^nd^ Ab** | **Manufacturer** |
| ***Primary antibody*** | | | | | | |
| Loricrin | FFPE | Rabbit | AF62 | 1:1000 | B | Covance, The Netherlands |
| Filaggrin | FFPE | Rabbit | PRB417 | 1:1000 | B | Covance, The Netherlands |
| Collagen type IV | FFPE | Mouse | 24.12.8 (PHM-12) | 1:75 | C | Chemicon, Australia |
| Laminin 332 | Frozen | Mouse | BM165 | 1:150 | C | Provided by Dr. M. Aumailley, Germany |
| Vimentin | FFPE | Mouse | V9 | 1:250 | C | Sigma-Aldrich, Germany |
| Alpha smooth muscle actin | FFPE | Mouse | 1A4 | 1:500 | C | Abcam, UK |
| ***Secondary antibody*** | | | | | | |
| B) Cy3-conjugated anti-rabbit |  | Goat |  | 1:500 |  | Jackson immunoresearch Laboratory, USA |
| C) Cy3-conjugated anti-mouse |  | Goat |  | 1:1000 |  | Jackson immunoresearch  Laboratory, USA |


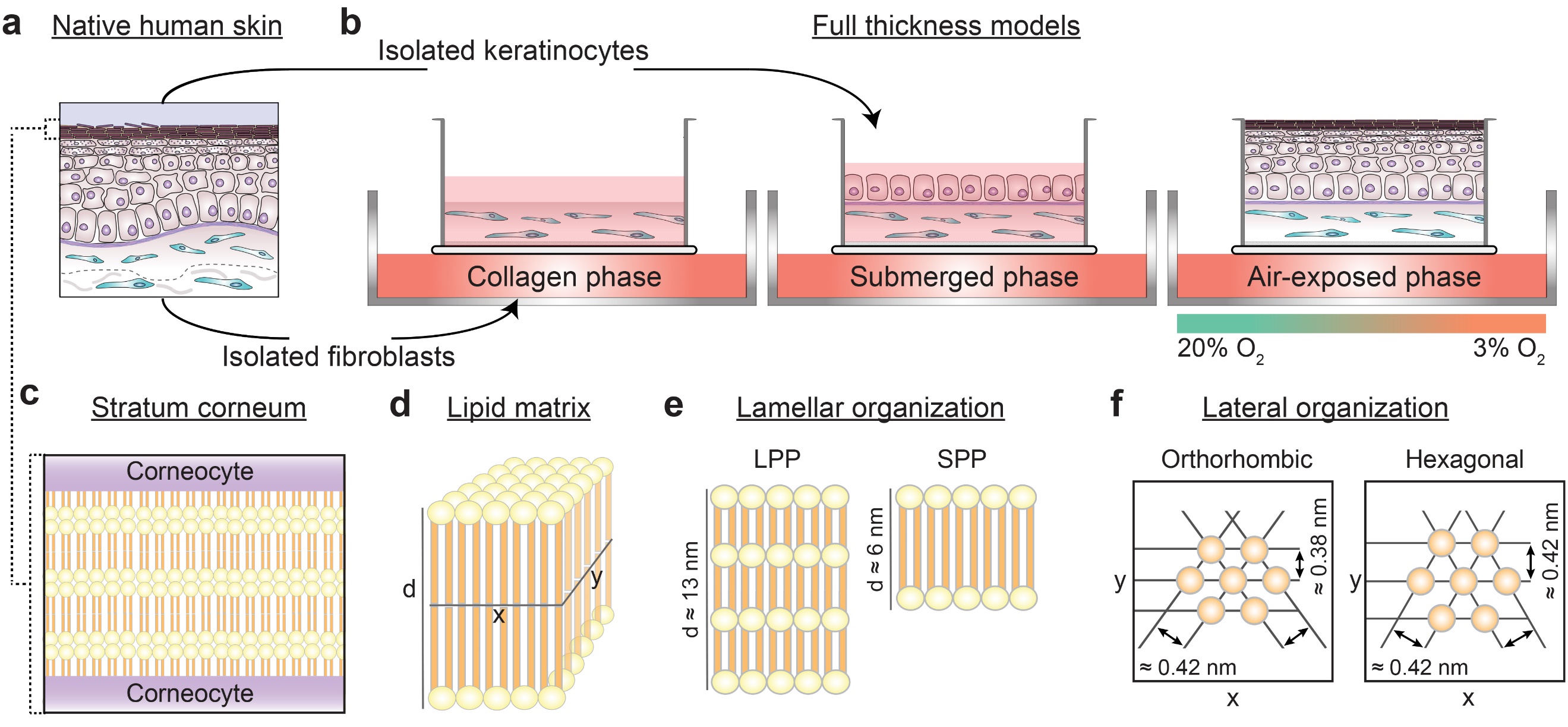


**Supplementary Figure 1.** **Lipid barrier in native human skin and human skin equivalents.** **(a)** Schematic overview of the dermis and epidermis of native human skin. **(b)** Development of full thickness models occurred in three main phases. Isolated primary fibroblasts were seeded in and rearranged the extracellular matrix during the collagen phase. Isolated primary keratinocytes were seeded on top of the collagen and proliferated during the submerged phase. Stratification occurred during air-exposed phase, at which oxygen levels were modulated in this study. **(c)** Schematic overview of the intercorneocyte lipid matrix of the stratum corneum. **(d)** Illustration of the lipid matrix. **(e)** Simplified overview of the lamellar organization. Long periodicity phase (LPP) and short periodicity phase (SPP) as shown by head group regions and hydrocarbon chain regions. Both LPP and SPP are characterized by a specific repeat distance^34^. **(f)** Lateral organization of the hydrocarbon chains of the lipids. The orthorhombic organization is a very dense ordered packing, whereas the hexagonal organization is less dense ordered.


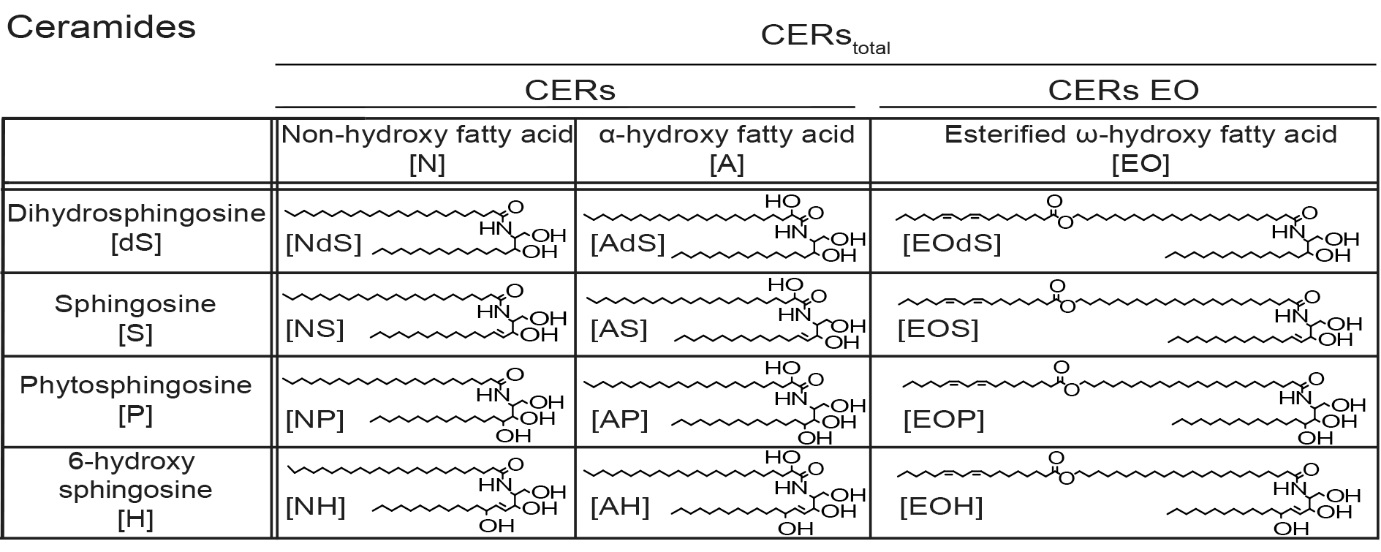


**Supplementary Figure 2.** **Ceramide subclasses of the lipid matrix.** Tabular overview of the structural formulas of twelve major ceramide subclasses, which consist of a sphingoid base coupled to a fatty acid chain. Total ceramides (CERs_total_) consist of CERs and CERs EO. The latter subclasses contain a characteristic esterified acyl chain. Ceramide nomenclature is followed according to
Motta *et al.*^63^.


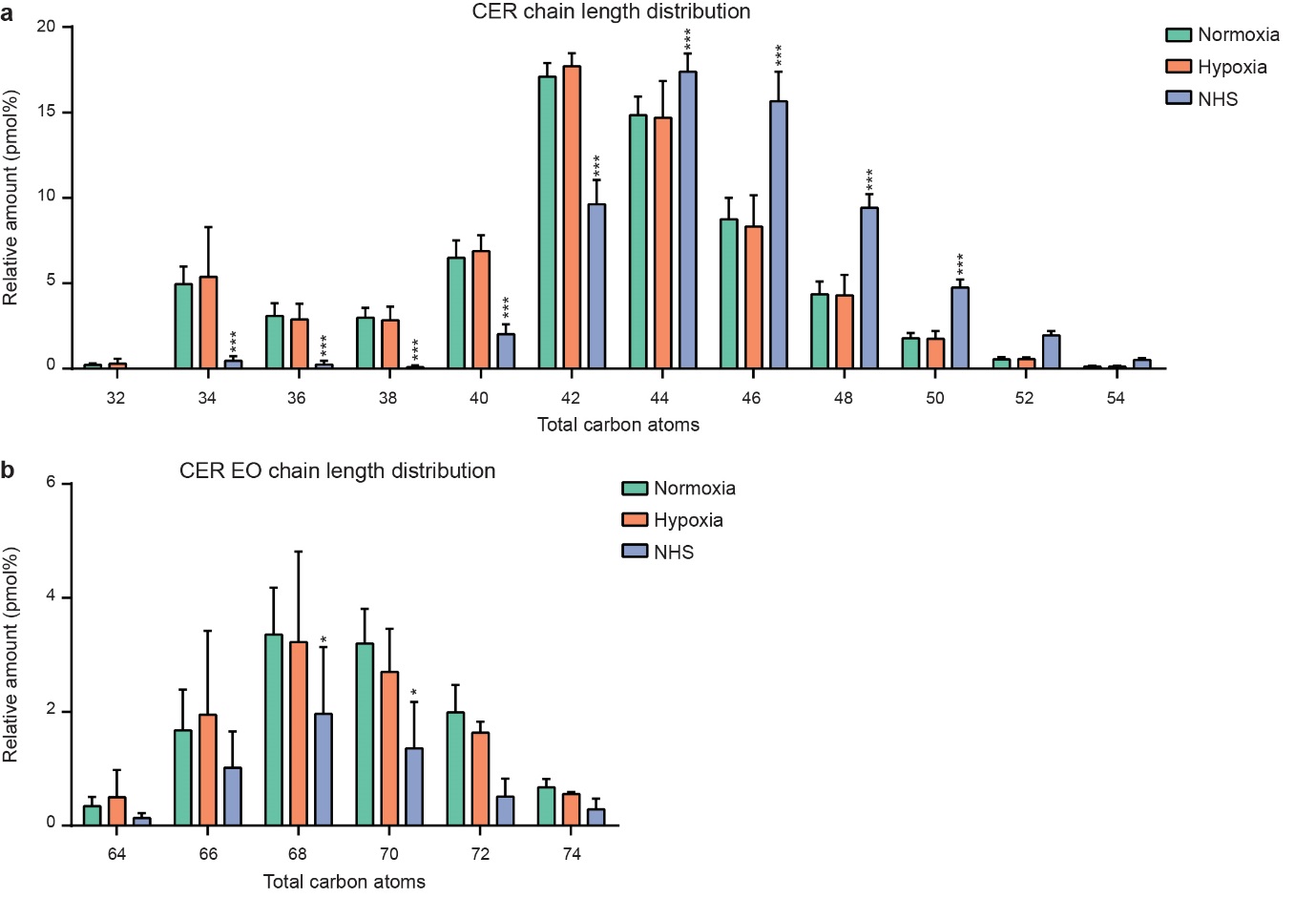


**Supplementary Figure 3.** **Stratum corneum CER and CER EO carbon chain length distribution**. **(a)** Bar diagram plot of CERs with an even number of carbon atoms of FTMs generated under normoxia or hypoxia and of NHS. **(b)** Bar diagram plot of CERs EO with an even number of carbon atoms of FTMs generated under normoxia or hypoxia and of NHS. All data represents mean + s.d.


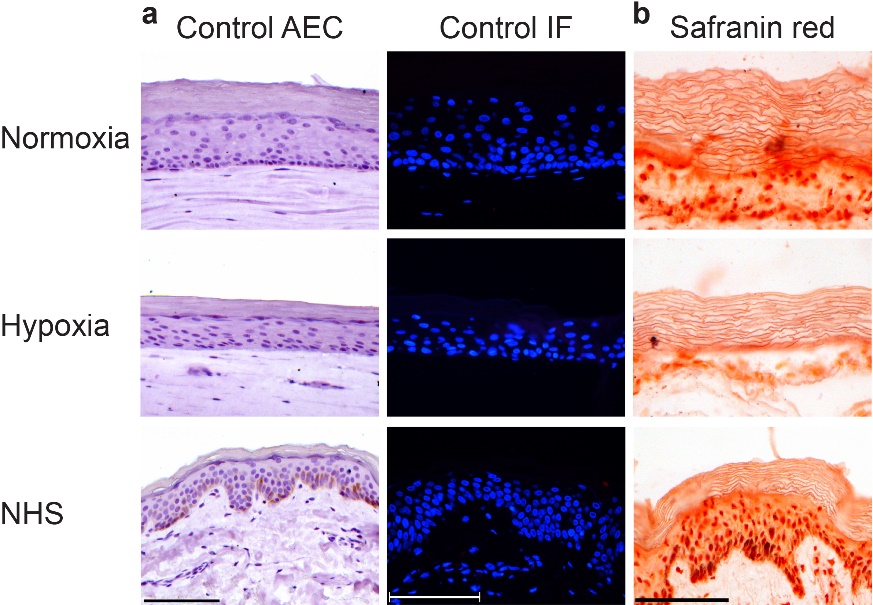


**Supplementary Figure 4.** **Safranin red staining and negative controls of immunohistochemistry.** **(a)** Representative cross sections of FTMs and NHS stained with safranin red followed by alkali expansion of the SC used for quantification of the number of corneocyte layers. **(b)** Negative controls for immunohistochemical and immunofluorescence stainings. Nuclei are stained blue using hematoxylin or DAPI. Scale bar indicates 100 μm.


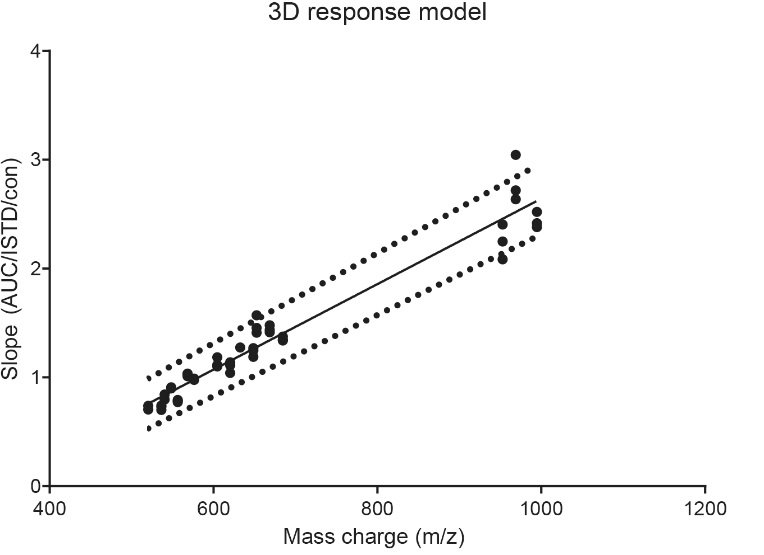


**Supplementary Figure 5.** **Three-dimensional response model used for quantification of the ceramide composition.** This 3D response model is used for quantification of the CERs_total_ composition. Model is based on mass spectrometry settings, compound properties, and a calibration curve from a limited number of synthetic ceramides, as described by Boiten *et al.*^62^.
